# A Preliminary Study Investigating Maternal Neurocognitive Mechanisms Underlying a Child-Supportive Parenting Intervention

**DOI:** 10.3389/fnbeh.2019.00016

**Published:** 2019-02-12

**Authors:** Nicole R. Giuliani, Kathryn G. Beauchamp, Laura K. Noll, Philip A. Fisher

**Affiliations:** ^1^Department of Special Education and Clinical Sciences, University of Oregon, Eugene, OR, United States; ^2^Center for Translational Neuroscience, University of Oregon, Eugene, OR, United States; ^3^Department of Psychology, University of Oregon, Eugene, OR, United States; ^4^Department of Psychological Sciences, Northern Arizona University, Flagstaff, AZ, United States

**Keywords:** strength-based parenting programs, video coaching, filming interactions to nurture development, inhibitory control, parenting self-evaluation

## Abstract

Although interventions that promote child-supportive parenting for children have been shown to positively impact caregiving behaviors as well as child behavioral and neurobiological functioning, less is known about which aspects of maternal brain functioning are affected by such interventions. In the present study, we conducted a preliminary evaluation of the impact of the Filming Interactions to Nurture Development (FIND) video coaching program on mothers with at least one child age four or younger. We employed a waitlist control design with pre-post data. Compared to mothers in the control condition (*n* = 16), mothers who received FIND (*n* = 16) showed changes in neural measures of inhibitory control and behavioral measures of parenting self-evaluation during a series of functional neuroimaging tasks. Specifically, we found a group by time interaction in clusters in the left inferior frontal gyrus (IFG) and insula for the Correct Stop > Correct Go contrast of the stop signal task (SST), where FIND increased brain activity associated with inhibitory control compared to mothers in the control condition; and FIND increased mothers’ endorsement of child-supportive parenting traits in the parenting self-evaluation task (PSET). Exploratory moderators, study limitations, and the implications of these findings for strength-based parenting programs are discussed.

## Introduction

Child-supportive parenting is a key determinant of positive cognitive, social, and emotional outcomes for children. Also known as warm, responsive, nurturing, contingent, and positive parenting in the literature, child-supportive parenting is characterized by a parent’s attunement to a child’s needs, as well as by their ability to provide positive reinforcement and consistent, non-harsh discipline when necessary (Pettit et al., 1997; Raby et al., 2015). Engagement in child-supportive parenting requires a number of skills on the part of the caregiver, including but not limited to the ability to recognize the child’s needs, and to regulate his or her own behavior, emotions, and actions in the service of providing support for the child (Volling et al., 2002). Notably, the importance of child-supportive parenting has been emphasized and investigated across a range of theoretical perspectives, from attachment and psychodynamic theory (Bernard et al., 2013) to social learning conceptualizations of development (Fisher and Skowron, 2017). The universality of supportive caregiving’s impact on healthy development among altricial mammalian species is also well documented in numerous rodent (Molet et al., 2014) and primate (Sanchez, 2017) models of biobehavioral development.

The presence of child-supportive parenting is a powerful buffer against the effects of social and economic adversity (Pettit et al., 1997; Lengua et al., 2015; Flannery et al., 2017). Such parenting is especially important during the first years of life, when behavioral and neurobiological systems are undergoing rapid development, and are strongly affected by environmental influences (Shonkoff et al., 2009). Individual differences in levels of child-supportive parenting are multi-determined by cultural values, prior life experiences (e.g., the models of parenting to which one has been exposed), and concurrent factors such as stress and Parenting Sense of Competence (PSOC; Raby et al., 2015). In addition, “risky” family characteristics such as higher levels of conflict, reduced support, and exposure to violence within the family (Repetti et al., 2002) and the presence among families in poverty of conditions such as food insecurity, inadequate housing, and neighborhood violence may understandably interfere with parental responsiveness in some individuals.

Interventions targeting child-supportive parenting have long been understood to be an effective intervention strategy to improve outcomes for children reared under conditions of adversity—particularly in infancy and early childhood (Dishion et al., 2008; Merz et al., 2016; Morris et al., 2017). This applies not only to commonplace experiences of adversity such as growing up in poverty, but also to children who experience severe forms of early life stress, such as institutional rearing (Nelson et al., 2014), as well as maltreatment, maternal separation, and placement in foster care (Bernard et al., 2015).

A number of evidenced-based interventions exist for enhancing child-supportive parenting among high-risk populations of infants and toddlers (Comer et al., 2013; Menting et al., 2013; Leijten et al., 2018). Some of these interventions have been disseminated widely in community-based settings in the United States and elsewhere, and have greatly increased the quality and consistency of services to families with young children in these communities (Webster-Stratton, 2014). Particularly noteworthy are interventions for which effects have been observed on children’s neurobiological systems such as the neuroendocrine stress-response system (Dozier et al., 2018). These programs provide important evidence that the deleterious impact of early life adversity on child development can be mitigated by interventions that foster corrective parenting experiences.

Although changes in: (a) supportive parenting behavior; (b) self-report of parenting stress and competence; and (c) child biobehavioral development have been documented in prior intervention studies, *much less is known about which aspects of parental brain functioning are affected by such interventions*. Given that the brain exhibits less structural and functional plasticity in adults than in young children, it may be that such changes in parents are less likely to occur. However, other interventions not focused on parenting (e.g., mindfulness meditation) have been shown to produce alterations in adult brain function and structure (Davidson et al., 2003; Hölzel et al., 2011; Weng et al., 2013; Kral et al., 2018) and longitudinal neuroimaging studies have documented dramatic brain changes that occur during the early post-partum period (e.g., Kim et al., 2010), suggesting there is much we do not yet understand about the plasticity of the parental brain. Moreover, the centrality of responsive caregiving in the basic survival of offspring might render it a viable candidate for promoting changes in adult brain functioning *via* intervention.

### Enhancing Child-Supportive Parenting With Video Coaching

One method that has proven useful in enhancing child-supportive parenting is video coaching (Mendelsohn et al., 2007). In the context of parenting interventions, caregivers are filmed during naturalistic interactions with their children and then view the films with a coach (Fukkink, 2008). Video coaching is used to show caregivers instances of themselves engaging in the behavior targeted by the intervention, often with the goal of increasing the frequency of those behaviors. Empirical studies have documented positive effects of video coaching on parenting behavior (for recent reviews, see Fukkink, 2008; Balldin et al., 2016). Much of this work has shown that the effects of video coaching interventions are contingent upon program duration, with shorter programs showing greater effectiveness in the behavioral domain (Fukkink, 2008). This is consistent with the “less is more” (Bakermans-Kranenburg et al., 2003) as well as the similar “short but powerful” (van IJzendoorn et al., 2005) hypotheses of prevention program efficacy.

The Filming Interactions to Nurture Development (FIND) video coaching program, which is the subject of the present study, is a brief video coaching program for caregivers of young children (Fisher et al., 2016). FIND focuses on enhancing patterns of infant-caregiver interactions known as “serve and return,” a metaphor developed at the Center on the Developing Child at Harvard University (2007) that describes attentive, responsive caregiving in accessible terms. Children naturally serve when they initiate interaction through gaze, vocalization, and action; and adults return the serve when they respond in child-supportive ways. With “serve and return” as its guiding framework, the overarching goal of FIND is to foster child-supportive parenting behavior by helping each caregiver recognize when they are attending to their child’s serves and, likewise, when they are responding to those serves in a developmentally-supportive manner. By reinforcing caregivers’ existing strengths within the context of a warm and supportive coaching session, FIND thus aims to increase the frequency of these child-supportive parenting behaviors and, in turn, support children’s development across domains.

The conceptual model underlying FIND (see Fisher et al., 2016) specifies caregiver-based targets of intervention, outcomes in the caregiver and child, and specific underlying neurocognitive capacities in the caregiver hypothesized to mediate the associations between targets and outcomes. However, the main effects of FIND on these hypothesized underlying mechanisms have not previously been evaluated. The goal of the present study was to investigate the magnitude of these effects, as a precursor to a larger clinical trial that would allow for a rigorous examination of parent neurocognitive mediators of FIND intervention effects.

### Candidate Neurocognitive Mechanisms Underlying FIND

We hypothesized that targeted behavioral training in child-supportive parenting through FIND would affect several cognitive capacities and associated neural substrates in caregivers. First, the serve and return model of FIND requires the caregiver to notice a child’s serve, return it in a child-supportive way, and then wait for the child to serve again. Each step of this process involves and reinforces caregiver executive function. This is especially true of inhibitory control, which is thought to underlie caregivers’ abilities to be perceptive, responsive, and flexible (Kienhuis et al., 2010). Specifically, in FIND, waiting for the child to serve again requires the caregiver to inhibit his or her prepotent desire to engage in a different activity and/or control the interaction with the child. As such, practicing this form of inhibitory control within the context of a supportive and behaviorally reinforcing intervention may positively impact caregivers’ underlying neurocircuitry. This hypothesis is consistent with recent work demonstrating more general effects of an attachment-based parenting intervention on decision-making neurocircuitry (Swain et al., 2017). Second, inasmuch as the intervention exclusively targets caregivers’ strengths (i.e., already existing supportive parenting behaviors), we examined whether FIND changed mothers’ own self-concept. Specifically, we focused on parenting self-evaluation as a means of operationalizing how caregivers think about themselves in their role as parents of young children.

#### Inhibitory Control

Appreciation for the impact of parental executive function on parenting behavior and in turn child development is growing, with calls for increased research in this area (Crandall et al., 2015). Recent work has increasingly demonstrated significant associations between parental executive function and parenting quality (Deater-Deckard et al., 2012; Meldrum et al., 2018), which may be moderated by parenting stress (Monn et al., 2017). In other words, parents with poor inhibitory control may be at higher risk for less child-supportive parenting, as parenting often requires controlling one’s prepotent response to challenging child behavior while under stress (e.g., frustration at a toddler tantrum after a long day)—leading some to theorize that inhibitory control may represent a key mechanism for the intergenerational transmission of negative parenting (Deater-Deckard et al., 2012; Bridgett et al., 2017). As such, it may be that the FIND intervention, by focusing on and positively reinforcing instances in which a parent inhibits their prepotent desire to control the situation (e.g., distract the tired toddler with a preferred activity) and instead waits for a child to serve again, improves caregivers’ inhibitory control.

#### Parenting Self-Evaluation

A large body of empirical work indicates that parental self-efficacy is associated with parental mental health, child-supportive parenting behavior, and child development (Bohlin and Hagekull, 1987; Teti and Gelfand, 1991; Coleman and Karraker, 2000; Jones and Prinz, 2005), all of which may have significant implications for care-supportive parenting and parent-child interactions. Consistent with this, low parenting self-efficacy has been identified in longitudinal research as a risk factor for negative dyadic interactions between parents and their children during early childhood (Verhage et al., 2013). In addition, associations between parental self-efficacy and children’s academic and social competence (Bogenschneider et al., 1997; Ardelt and Eccles, 2001; Junttila et al., 2007) highlight the downstream implications of such negative interactions.

Taken together, this large and growing literature suggests that there may be two categories of self-evaluations that caregivers make about their own parenting behavior: developmentally supportive and developmentally unsupportive. Supportive traits promote serve and return interactions, such as “attentive,” “encouraging,” and “responsive.” In contrast, unsupportive traits act in opposition of these interactions, such as “distracted,” “exhausted,” and “overwhelmed” (Noll et al., 2018). As such, it may be that, by showing caregivers brief video clips of themselves naturally engaging in child-supportive interactions with their children, FIND increases their positive parenting self-evaluations and decreases their negative ones.

### The Current Study

The primary purpose of the current study was to examine the impact of the FIND program on mothers of young children with regard to hypothesized impacts on parenting perceptions and underlying neurocognitive mechanisms. Consistent with the FIND “theory of change” (Fisher et al., 2016), which describes multiple underlying neurocognitive capacities through which the elements of FIND (i.e., intervention targets) impact outcomes across domains (e.g., supportive parenting, parenting stress), we predicted that, compared to mothers in a waitlist control group, mothers receiving FIND would demonstrate improvements in maternal function across several domains: self-reported parenting stress and self-efficacy, and behavioral and neural indices of inhibitory control and parenting self-evaluation. We additionally explored the moderating role of family income, the age of the child targeted by the FIND intervention, and the mother’s history of childhood adversity, age, and number of children.

## Materials and Methods

### Participants

Mothers with at least one child age four or under were recruited *via* fliers posted in the community and targeted advertising on social media. A total of 37 mothers came in for the initial MRI scan, after which they were assigned to either intervention (FIND program; EXP) or control (waitlist; CTL) groups. These 37 mothers are the sample explored in Noll et al. (2018). As such, some of the methods described below overlap with those methods, and are noted as such. Of these 37 mothers, 32 returned for their follow-up MRI scan after completing FIND or waiting the equivalent amount of time. These mothers ranged in age from 20 to 43 years (*M* = 31.22, *SD* = 5.615), and their racial/ethnic composition was representative of the region: 90.6% Caucasian, 6.3% Hispanic, and 3.1% Asian/Pacific Islander. Maternal education ranged from General Education Diploma through to doctoral diploma, and family gross income ranged from $0 to $200,000 per year (*M* = $54,390, *SD* = $37,672). The age of the target child (mother’s youngest biological child age four or under) ranged from 7 weeks to 4 years (*M* = 1.66 years, *SD* = 1.25). Approximately half of mothers in each group were primiparous (7 EXP, 6 CTL), and the total number of children ranged from one to six (*M* = 1.97, *SD* = 1.26) across the whole sample. The mothers who did not return for their second MRI scan (3 EXP, 2 CTL) were not significantly different than the ones who did with regard to age, education, income, age of target child, number of children, childhood adversity, or self-reported parenting stress, parenting self-efficacy, or mood (*p*-values > 0.30). These demographic characteristics also did not differ for the two groups of participants who completed the full study (*p*-values > 0.29). Full sample demographics and self-report variables gathered at baseline are listed in Table 1.

**Table 1:** Means and standard deviations (SDs) among demographic and self-report variables reported at baseline (T1) by group.

	Group					
	FIND (*n* = 16)		Control (*n* = 16)		Full sample (*N* = 37)	
	*M*	*SD*	*M*	*SD*	*M*	*SD*
Age	32.60	5.39	30.87	5.85	31.72	5.81
Income	65.04	64.03	54.63	27.99	61.26	49.65
Education	15.50	2.92	14.69	2.33	14.95	2.61
Age of TC	1.98	1.19	1.54	1.59	1.72	1.35
Number of children	2.00	1.32	1.94	1.24	1.89	1.20
ACES	3.38	2.73	2.31	2.90	2.97	2.85
PSI-TOT	73.25	18.33	75.44	12.17	74.43	15.74
PSI-PD	27.00	8.12	29.19	6.29	28.08	7.55
PSI-PCDI	19.39	6.14	20.25	4.77	20.09	5.51
PSI-DC	26.96	6.66	26.00	3.83	26.31	5.53
PSOC	53.06	5.36	52.00	4.63	52.43	5.59
PA	36.63	7.56	34.63	8.03	36.14	7.58
NA	19.31	6.69	20.69	7.30	20.05	6.75

*Note. Filming Interactions to Nurture Development (FIND) and Control groups include participants who completed both sessions. Age, maternal age (years); Income, annual household gross income (thousands of dollars/year); Education, number of years; Age of TC, age of target child (years); ACES, Adverse Childhood Experiences Scale Total Score; PSI–TOT, Parenting Stress Index Total Score; PD, Parental Distress Subscale; PCDI, Parenting-Child Dysfunctional Interaction; DC, Difficult Child; PSOC, Parenting Sense of Competence Total Score; PA, Positive Affect Subscale Score from the PANAS; NA, Negative Affect Subscale Score from the PANAS*.

### Procedure

All procedures utilized in the current study were approved and monitored by the University’s Office for the Protection of Human Subjects. Mothers of young children were recruited from an urban area in the Pacific Northwest and screened by phone for eligibility. Inclusion criteria included having at least one biological child within the target age range, the absence of neurological disorders known to image MRI measures, the absence of standard MRI contraindications, and right-handedness. Following recruitment and screening, eligible participants were scheduled for an initial MRI session at the University’s Neuroimaging Center, which consisted of informed consent, an MRI session, and self-report questionnaires At the end of this initial session (T1), participants were sequentially assigned to FIND or control groups based on FIND coach availability. No information about the families was used to assign them to group, and groups did not differ with regard to demographics (*p*-values > 0.29) or baseline measures of affect, stress, or self-efficacy (*p*-values > 0.4). After completion of the FIND program or the equivalent amount of time, participants were invited back for their follow-up MRI session (T2), which was identical to the first.

### Conditions

#### FIND

The FIND video coaching program typically targets five child-supportive parenting behaviors in a structured sequence across 10 30–45-min sessions that alternate between filming and coaching. Each parenting behavior is based on the core concept of “serve and return,” and builds on the one before: (1) sharing the child’s focus; (2) supporting and encouraging; (3) naming (a specific kind of supporting and encouraging); (4) back-and-forth; and (5) endings and beginnings. The process begins with an initial visit during which the coach provides an overview, records 10–15 min of the caregiver and child engaged in everyday interactions, then introduces the concept of serve and return. The video is edited to contain brief clips in which the caregiver is engaged in the first of five specific and precisely defined caregiver-based components of serve and return. The next week, the FIND coach reviews the edited film with the caregiver in a systematic manner previously described by Fisher et al. (2016). Continuing sessions alternate between filming and coaching sessions until all five components have been covered sequentially (one session per week for a total of 10 weeks).

#### Waitlist Control

Families assigned to the waitlist control group were informed of their group assignment after the completion of the T1 session. They were told that they would be contacted in about 3 months for their follow-up session, and that they should not participate in any parenting programs or interventions in the interim. After T2, they were invited to receive the FIND intervention.

### Measures

#### Self-Report Measures

Mother’s self-reported experience of parenting stress, self-efficacy, and adversity were measured consistent with the procedures described by Noll et al. (2018). Specifically, parenting stress was measured using the Parenting Stress Index, Third Edition Short Form (PSI-3-SF; Abidin, 1990), which contains subscales assessing parental distress, parent-child dysfunctional interaction, and difficult child (DC). Parenting self-efficacy was measured using a modified version of the PSOC (Johnston and Mash, 1989). State affect was measured using the Positive and Negative Affect Schedule (PANAS; Watson et al., 1988). Participants’ childhood history of adversity was measured using an abbreviated version of the Adverse Childhood Experiences Scale (ACES; Felitti et al., 1998). Basic demographic information was collected *via* a questionnaire created by the researchers. All surveys were administered at the first session after the MRI tasks, and the PSI-3-SF, PSOC, and PANAS were administered again at the second session after the MRI to test for group by time interactions on self-reported parenting stress and parenting self-efficacy, controlling for affect.

#### Stop Signal Task

Mothers’ inhibitory control was assessed using the stop signal task (SST; Verbruggen and Logan, 2008). As described by Berkman et al. (2014), who recently utilized this paradigm in their study of inhibitory control training, each trial of the SST consists of a cue (500 ms), followed by an arrow pointing either left or right (with 1:1 relative frequency) that serves as the go signal (1,000 ms), and then an inter-trial interval of variable duration (*M* = 1,400 ms; jittered following a gamma distribution). Participants are instructed to press the left or right arrow key as quickly as possible in response to the go signal. On 25% of the trials, an auditory stop signal is played after the go signal at a variable latency known as the stop-signal delay (SSD). Participants are instructed to withhold their button press on trials in which a stop signal sounded. The SSD is adjusted by 50 ms after each stop trial using a staircase function that increases for successful stops and decreases for failed stops. Two independent staircases alternate control over the SSD in blocks of eight trials until 50% response accuracy is reached on stop trials. The critical measure, the stop-signal response time (SSRT), is an index of the efficiency of the inhibitory control process. The SSRT was calculated as the difference between the speed of the stop process and the SSD. As in previous work using this paradigm (e.g., Berkman et al., 2014) and per the recommendations of Verbruggen et al. (2013), we employed an integration method to estimate the speed of the stop process (i.e., identifying the point at which the integral of the distribution of reaction times equals the probability of responding given a specific delay). Each run consisted of 128 trials (32 stop trials) and lasted 6:06 min. The SSRT was computed separately for each run and averaged across the two runs, which then was used as the main index of behavioral performance on the SST.

#### The Parenting Self-Evaluation Task (PSET)

Mothers’ self-identification with positive and negative parenting qualities was assessed using the parenting self-evaluation task (PSET; Noll et al., 2018). As described by Noll et al. (2018), in the PSET, participants are presented with positively- and negatively-valenced terms that index child-supportive (“DS”) or developmentally unsupportive (“DU”) caregiving behavior, respectively. Blocks vary by instruction, asking participants to evaluate whether these words described them as a parent (Self) or, conversely, whether they believe these qualities can change for parents in general (Change). In the current study, multiparous mothers were instructed to respond to self-evaluate themselves as parents with respect to the study’s target child.

The PSET paradigm is a mixed block/event-related design, consisting of two block types representing evaluation perspective and two event types representing trait valence. This produces a total of four conditions, with 26 trials per condition. For each trial, participants answer the prompt *via* a left or right button press indicating a “yes” or “no” response. Behavioral performance on the PSET is calculated as percent of qualities endorsed in each condition. As patterns associated with reaction time were shown to follow those associated with percent of qualities endorsed, we focus here on the latter to keep the number of variables included in our analyses to a minimum.

### fMRI Data Collection and Analysis

As described in Noll et al. (2018), data in the current study were acquired using a 3.0 Tesla Siemens Skyra scanner at the LCNI. Blood oxygen-level dependent echo planar images (BOLD-EPI) were acquired with a T2*-weighted gradient echo sequence (TR = 2,000 ms, TE = 25 ms, flip angle = 90, matrix size = 104 × 104, 72 contiguous axial slices with interleaved acquisition, field of view = 200 mm, slice thickness = 2 mm; total time = 5 min 50 s per run × 2 runs for PSET; total time = 6 min 6 s per run × 2 runs for SST). For each participant, a high-resolution structural T1-weighted 3D MPRAGE pulse sequence (TR = 2,300 ms, TE = 2.1 ms, matrix size = 192 × 192, 160 contiguous axial slices, voxel size = 1 mm, slice thickness = 1 mm; total time = 5 min 59 s) was acquired coplanar with the functional images, as well as a pair of opposite phase encoded images (SE-EPI) to be used to account for inhomogeneities in the magnetic field within the functional images (TR = 6,390 ms, TE = 47.8 ms, flip angle = 90, matrix size = 104 × 104, 72 slices, field of view = 200 mm, slice thickness = 2 mm, total time = 1 min 8 s per run × 2 runs). Task order was counterbalanced across subjects; two additional functional runs were collected that are not reported here.

Before preprocessing, all DICOM images were converted to NIfTI format *via* MRI-Convert^1^, and non-brain tissue was removed from MPRAGE images using robust skull stripping with the Brain Extraction Tool in FMRIB’s Software Library (FSL)^2^. All further analyses were conducted using SPM12 (Wellcome Department of Cognitive Neurology, London, UK)^3^. Briefly, MPRAGE images were coregistered to the MNI template and segmented into gray matter, white matter, and cerebrospinal fluid, and combined to create a study-specific template using the DARTEL toolbox for SPM12. Field inhomogeneities were corrected by using a fieldmap to unwarp functional images. Images were motion-corrected using realignment, and the mean of all functional images was co-registered to each subject’s own structural MPRAGE using a six-parameter rigid body transformation model. All images were then spatially normalized into MNI template space using the study-specific template, and smoothed using a 4 mm^3^ full-width at half-maximum Gaussian kernel.

Statistical analyses were conducted in SPM12. For each subject, event-related condition effects were estimated separately for each task according to the general linear model, using a canonical hemodynamic response function, high-pass filtering (128 s) and a first-order autoregressive error structure. BOLD signal was then modeled in a fixed effects analysis with separate regressors modeling each condition of interest (SST model: Correct Go, Correct Stop, Incorrect Stop, Cue phase; PSET model: Self DS, Self DU, Change DS, Change DU for the first 4 s of each trial). Five-parameter motion regressors were calculated as deviations from the origin (Euclidean translation, Euclidean rotation, derivative of Euclidean translation, derivative of Euclidean rotation, and trash), and entered into single-subject models as covariates of non-interest. Button press (left or right index finger) and reaction time were also included as covariates of non-interest in the single-subject models for the PSET. Linear contrasts were created for the contrasts of interest for each task for each participant (SST: Correct Stop > Correct Go; PSET: Self > Change) over time (T2 > T1). We chose to model the effect of time at the single-subjects level because change over time takes place within-subjects. These contrasts were then imported to group-level independent subjects *t*-tests, where main effects were modeled for inference to the population separately by task.

Since the brain regions previously identified in both the SST and PSET encompass large cortical structures, we decided to interrogate individual differences in engagement of specific regions of interest (ROIs) for each task. For the SST, we decided to build anatomical ROIs in three of the main regions of the inhibitory control network, the left inferior frontal gyrus (IFG), right IFG, and dorsal anterior cingulate cortex (dACC; Aron et al., 2007; Swick et al., 2008; Munakata et al., 2011). The left and right IFG ROI was also built using the WFU PickAtlas (aal labels Frontal_Inf_Tri_L and Frontal_Inf_Tri_R), and the dACC ROI was built using the procedure detailed in Cascio et al. (2015). The PSET recruits a broad swath of cortical midline structures (Noll et al., 2018), including the medial prefrontal cortex (mPFC). As in previous work (Noll et al., 2018), since several of our hypotheses were specific to mPFC activity associated with self-evaluation, we utilized an anatomical ROI based on the WFU Pickatlas anterior cingulate volume (TD label Anterior Cingulate; Maldjian et al., 2003), which overlaps with clusters found in previous investigations of self-evaluation (see Jankowski et al., 2014). Parameter estimates of individual subjects’ activity in these ROIs for the change in the contrast of interest over time were extracted from SPM beta images using MarsBar (MRC Cognition and Brain Sciences Unit, Cambridge, UK)^4^ for analyses detailed below.

### Analytic Approach

We computed descriptive statistics for task performance (SSRT; PSET percent endorsed and reaction time across all qualities and by valence), as well as self-reported parenting stress, parenting self-efficacy, and positive and negative trait affect at both time points. Individual averages of BOLD signal in the ROIs (left and right IFG and dACC for the SST contrast; mPFC for the PSET contrast) were exported to SPSS (version 24.0, IBM) for further analyses. For all variables, outliers were winsorized at 3 standard deviations (SDs) from the mean, and checked for normality. Variables found to be significantly skewed were transformed to improve normality, as indicated in the “Results” section. For behavioral and self-report variables, change was calculated in SPSS as value at T2 minus value at T1. Hypothesized group by time effects on self-report and behavioral variables were interrogated using repeated measures analysis of variance (rmANOVA) models, with covariates noted for each test. Hypothesized group effects on fMRI parameter estimates were interrogated using multiple linear regression models.

We additionally individually explored the role of hypothesized moderators of these effects, which include ACES, maternal age, number of children, age of the child targeted by the intervention, family income, and maternal education. Models investigating moderations on self-report and behavioral variables were run using rmANOVAs. As the effect of time on brain activity was calculated at the single-subject level for SST and PSET models, the parameter estimates we extracted contained the effect of time for each contrast of interest. Therefore, moderation of these effects was explored using analyses of covariance (ANCOVA), testing the effect of group on the change over time.

## Results

### Self-Report Measures

Means and SDs of self-reported PSOC (as measured by the PSOC), parenting stress (as measured by the PSI-3-SF), and positive and negative affect (as measured by the PANAS) by group and time point are listed in Table 2. At baseline, total self-reported parenting stress was significantly associated with positive affect (*r*_(37)_ = −0.6, *p* < 0.001) and negative affect (*r*_(37)_ = 0.753, *p* < 0.001) in the expected directions. The two groups were not significantly different on any of the self-report measures (*p*s > 0.4). Across all participants, change in parenting stress from T1 to T2 was significantly correlated with change in PA (*r*_(32)_ = −0.446, *p* = 0.011) and change in NA (*r*_(32)_ = 0.363, *p* = 0.041). Neither baseline nor changes in self-reported PSOC were significantly associated with either baseline or changes in PA or NA (*p*s > 0.25).

**Table 2:** Means and SDs among variables gathered by time and group.

	T1				T2			
	FIND		Control		FIND		Control	
	*M*	*SD*	*M*	*SD*	*M*	*SD*	*M*	*SD*
PSI-TOT	73.25	18.33	75.44	12.17	74.65	20.06	77.25	15.05
PSI-PD	27.00	8.12	29.19	6.29	29.06	9.97	29.75	5.87
PSI-PCDI	19.39	6.14	20.25	4.77	19.43	5.95	21.13	4.81
PSI-DC	26.96	6.66	26.00	3.83	26.50	6.88	26.38	6.13
PSOC	53.06	5.36	52.00	4.63	37.06	8.13	36.44	5.16
PA	36.63	7.56	34.63	8.03	36.44	6.40	35.94	7.57
NA	19.31	6.69	20.69	7.30	18.56	7.04	20.13	7.94
SSRT	273.50	68.32	254.28	49.78	230.94	72.29	254.64	59.59
PSET %DS-S	86.31	15.71	93.74	7.95	93.42	10.05	94.19	7.06
PSET %DU-S	20.11	15.10	23.45	10.87	14.48	13.79	17.90	8.38
PSET %DS-C	83.63	23.07	86.17	19.32	86.79	16.34	87.07	20.03
PSET %DU-C	78.62	21.49	88.32	10.28	77.72	23.21	87.78	15.94

*Note. T1, Time 1 (baseline); T2, Time 2 (follow-up); FIND (*n* = 16) and Control *(n =* 16) groups included participants who completed both sessions, except *n* = 15 for Control group T2 SSRT. PSI-TOT, Parenting Stress Index Total Score; PD, Parental Distress Subscale; PCDI, Parenting-Child Dysfunctional Interaction; DC, Difficult Child; PSOC, Parenting Sense of Competence Total Score; PA, Positive Affect Subscale Score from the PANAS; NA, Negative Affect Subscale Score from the PANAS; SSRT, stop signal reaction time as measured from the stop signal task; PSET, Parenting Self Evaluation Task; PSET %DS-S, percentage of child-supportive parenting qualities endorsed during the PSET in the “Self” condition; PSET %DU-S, percentage of developmentally unsupportive parenting qualities endorsed during the PSET in the “Self” condition; PSET %DS-C, percentage of child-supportive parenting qualities endorsed during the PSET in the “Change” condition; PSET %DU-C, percentage of developmentally unsupportive parenting qualities endorsed during the PSET in the “Change” condition*.

Group by time interactions from the rmANOVAs for self-reported PA and NA were not significant (*p*s > 0.46). Similarly, PSOC and parenting stress (total and subscales) did not show significant group by time interactions (*p*s > 0.45).

### Stop Signal Task

#### Behavior

**Figure 1:**
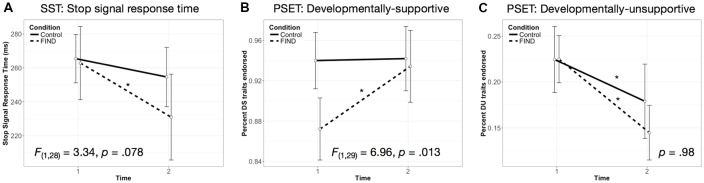
Group by time effects on behavior during the stop signal task (SST) and parenting self-evaluation task (PSET). The Filming Interactions to Nurture Development (FIND) group showed **(A)** a significant decrease in stop-signal response time (SSRT), *F*_(1,28)_ = 8.53, *p* = 0.007, not seen in the CTL group, *p* = 0.8, **(B)** a significant increase in endorsement of DS traits, *F*_(1,29)_ = 12.45, *p* = 0.001, not seen in the CTL group, *p* = 0.82, and **(C)** a significant decrease in endorsement of DU traits over time, *F*_(1,29)_ = 4.87, *p* = 0.035, which the CTL group also showed, *F*_(1,29)_ = 5.07, *p* = 0.032. Note: *p < 0.05. Error bars indicate 95% confidence interval.

Performance on both tasks is listed by group and time point in Table 2. At baseline, the two groups did not show significantly different SSRTs, *F*_(1,31)_ = 0.826, *p* = 0.371. A rmANOVA revealed a trending group by time interaction on the SSRT, *F*_(1,29)_ = 3.88, *p* = 0.059 (Figure 1A). Due to the preliminary nature of this study, we chose to perform follow-up simple effects. These analyses revealed that the FIND intervention group showed a significant decrease in SSRT (i.e., improvement in inhibitory control) between T1 (*M* = 273.50 ms, *SD* = 63.32) and T2 (*M* = 230.95 ms, *SD* = 72.30), *F*_(1,29)_ = 8.78, *p* = 0.006, whereas the CTL group did not (*p* = 0.9).

#### Brain

**Figure 2:**
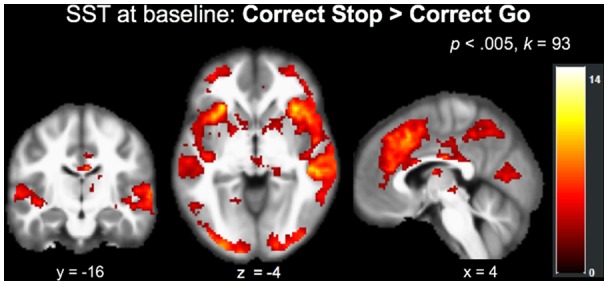
Recruitment of regions implicated in inhibitory control during the SST (Correct Stop > Correct Go) at baseline across the entire sample (*N* = 37).

The main effect of stopping in the Correct Stop > Correct Go contrast at baseline (*N* = 37, collapsed across group) at an FWE-corrected (*p* < 0.05) cluster-threshold of *k* = 93 (*p* < 0.005) revealed a network of regions implicated in inhibitory control, including but not limited to right insula, left IFG, right posterior cingulate and precuneus, and left cerebellum (Table 3; Figure 2).

**Table 3:** Peak voxel and maximum *T*-values for SST baseline and group by time effects.

				MNI coordinates		
Region	Cluster size	*T*-statistic	Side	*x*	*y*	*z*
**Baseline: Correct Stop > Correct Go**						
Insula	30,461	14.79	Right	32	24	4
Inferior frontal gyrus	-	11.91	Left	−34	18	−6
Middle temporal gyrus	-	11.03	Right	64	−38	4
Posterior cingulate cortex	1,497	8.04	Right	4	−16	28
Precuneus	-	6.95	Right	12	−70	40
Cerebellum	1,177	7.54	Left	−14	−74	−24
Precuneus	999	7.20	Right	18	−96	2
Interior occipital gyrus	-	6.58	Right	38	−88	−2
Fusiform gyrus	-	4.25	Right	42	−70	−14
Parahippocampal gyrus	129	5.33	Right	20	−24	−8
Cerebellum	97	4.39	Right	38	−58	−32
**Group by Time: FIND > Control (T2 > T1, Correct Stop > Correct Go)**						
Temporal pole	169	5.17	Left	−52	4	−8
Inferior frontal gyrus	-	4.46	Left	−30	16	−10
Insula	92	4.17	Left	−36	12	2

*Note: baseline: N = 37; p < 0.005, k = 93 (FWE-corrected p < 0.05). Group by Time: N = 32, p < 0.005, k = 82 (FWE-corrected p < 0.05)*.

**Figure 3:**
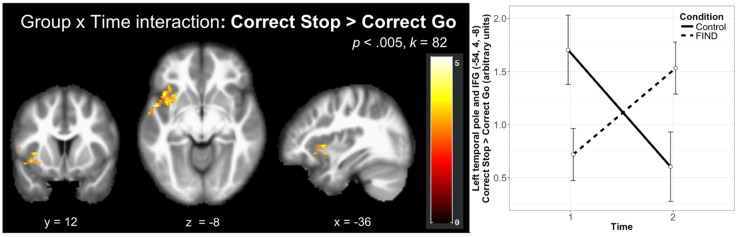
Group by time effects on brain activation during the SST revealed significant clusters in the left temporal pole extending into the left inferior frontal gyrus (IFG) and the left insula that were driven by increases in these regions in the FIND group and decreases in the CTL group over time (*N* = 32). This effect is decomposed by group and time on the left panel. Error bars indicate 95% confidence interval.

The whole-brain investigation of the effect of time by group (T2 > T1, Correct Stop > Correct Go) at a FWE-corrected (*p* < 0.05) cluster-threshold (*k* = 82, *p* < 0.005) revealed two clusters in the left hemisphere, in the left temporal pole extending into the IFG (*k* = 169) and the left insula (*k* = 92; Table 3). As shown in Figure 3, decomposition of these interactions by group and time demonstrated that these effects were driven by an increase in activity over time in the FIND group, but a decrease in the control group. Follow-up anatomical ROI analyses did not reveal significant effects of group on change over time in Correct Stop > Correct Go activity in the left IFG (*p* = 0.18), right IFG (*p* = 0.35), or dACC (*p* = 0.44). Within the left IFG ROI, however, there was a trend-level effect of group on the association between change in Correct Stop > Correct Go activity and change in self-reported PSOC (*F*_(1,27)_ = 3.27, *p* = 0.082). Follow-up regression analyses by group revealed that the control group had a non-significant negative association between change in PSOC and change in left IFG activity (*p* = 0.3) while the FIND group had a non-significant positive association (*p* = 0.22).

### Parenting Self-Evaluation Task

#### Behavior

At baseline, the two groups did not show significantly different percent endorsement of DS, *F*_(1,31)_ = 2.9, *p* = 0.102, or DU traits, *F*_(1,31)_ = 0.52, *p* = 0.47. A rmANOVA revealed a significant group by time interaction on the percentage of child-supportive traits (%DS) endorsed by participants, *F*_(1,30)_ = 4.418, *p* = 0.044 (Figure 1B). Follow-up simple effects analyses revealed that the FIND intervention group showed a significant increase in %DS between T1 (*M* = 0.86, *SD* = 0.16) and T2 (*M* = 0.93, *SD* = 0.1), *F*_(1,30)_ = 10.05, *p* = 0.003, whereas the CTL group did not (*p* = 0.84). For the rmANOVA modeling the percentage of developmentally unsupportive traits (%DU) endorsed by participants, both groups showed a significant decrease in %DU traits between T1 and T2 (FIND: *F*_(1,30)_ = 5.72, *p* = 0.023; CTL: *F*_(1,30)_ = 5.56, *p* = 0.025), with no significant group by time interaction (*p* = 0.98; Figure 1C).

#### Brain

**Figure 4:**
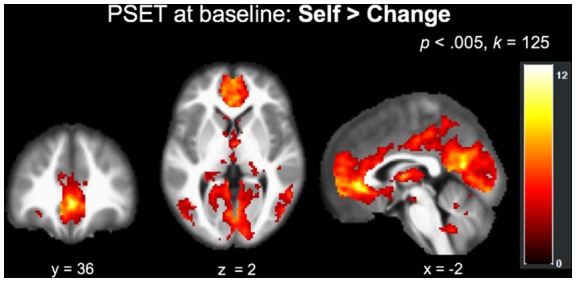
Recruitment of regions implicated in self-processing during the PSET (Self > Change) at baseline across the entire sample (*N* = 37).

The main effect of Self > Change across both types of traits at baseline (*N* = 37, collapsed across group) at an FWE corrected (*p* < 0.05) cluster threshold of *k* = 125 (*p* < 0.005) revealed a network of regions implicated in self-reflective processing (Figure 4), including but not limited to the left precuneus, bilateral ventral anterior cingulate and mPFC, left middle occipital gyrus, left and right cerebellum, and left insula extending into the inferior parietal lobule (Table 4).

**Table 4:** Peak voxel and maximum *T*-values for PSET baseline (T1).

				MNI coordinates		
Region	Cluster size	*T*-statistic	Side	*x*	*y*	*z*
**Baseline: Self > Change**						
Precuneus	22,013	12.56	Left	−4	−64	18
Middle orbital gyrus	-	11.33	Left	−2	36	−2
Thalamus	-	9.11	Left	0	−6	4
Middle occipital gyrus	1,046	6.05	Left	−36	−74	32
Middle temporal gyrus	-	5.71	Left	−50	−64	8
Inferior occipital gyrus	-	3.01	Left	−40	−86	2
Cerebellum	148	5.42	Left	−28	−82	−34
Cerebellum	601	5.32	Left	−4	−56	−46
Cerebellum	-	5.03	Right	14	−46	−46
Cerebellum	-	4.01	Left	−20	−40	−50
Precentral gyrus	190	5.29	Left	−26	−24	54
Insula	164	4.33	Left	−42	−30	24
Inferior parietal lobule	-	3.17	Left	−64	−24	28

*Note: N = 37; p < 0.005, k = 125 (FWE-corrected p < 0.05)*.

The whole-brain investigation of the effect of time by group (T2 > T1, Self > Change across all traits) did not reveal any significant clusters above the FWE-corrected (*p* < 0.05) cluster-threshold of *k* = 109 (*p* < 0.005). Follow-up anatomical ROI analyses did not reveal significant effects of group on change in Self > Change activity over time in the mPFC (*p* = 0.96). There were no group effects on the association between activity in this ROI and any of the self-report measures (*p* = 0.23). *Post hoc*, we re-ran analyses on just the Self > Change evaluations of DS traits to see if perhaps the null behavioral findings for DU traits were washing out any effects of FIND on brain activity associated with DS trait judgment, but these results were similarly null. We also tested to see if baseline levels of mPFC activity for the Self > Change contrast for DS traits predicted change in self-report. Indeed, across all participants, baseline activity in the mPFC ROI showed a trend-level association with change in self-reported parenting stress (*r*_(32)_ = −0.346, *p* = 0.052). This was mainly driven by the Parent-Child Difficult Interaction (PCDI) subscale (*r*_(32)_ = −0.424, *p* = 0.016), and suggests that, independent of intervention group, lower baseline mPFC activity associated with thinking about one’s own DS parenting traits predicted greater changes in self-reported difficult parent-child interactions.

### Exploratory Moderation Analyses

We explored the moderating effect of several key demographic and individual difference variables on the dependent measures interrogated in this study. In particular, we were interested in how family variables (i.e., maternal age, maternal education, maternal ACES, number of children, age of the child targeted in the FIND intervention, and family income) influenced change in self-reported parenting stress, PSOC, and SST and PSET performance and associated brain activity. It is important to note that most of these variables were positively skewed (i.e., age of the child targeted by the FIND intervention, number of children, family income, maternal education, and maternal ACES), and that transformation (square root) only improved the distribution of income. We decided to retain the original distribution of all other family variables, and interpret the results with caution. As noted in the “Materials and Methods” section, all moderation analyses were performed using rmANOVAs except for those investigating brain activity, which were performed using ANCOVAs.

Maternal history of childhood adversity (ACES) showed a moderation of the group effect on change in self-reported parenting stress specific to difficult parent-child interactions, *F*_(1,28)_ = 5.24, *p* = 0.03. Follow-up regression models by group revealed that the FIND mothers showed a trend-level negative association between ACES and change in PCDI (*t* = −2.076, *p* = 0.058) and the control group did not (*t* = 1.363, *p* = 0.196). We additionally found that maternal age significantly moderated the group effect of FIND on change in self-reported parenting sense of competency, *F*_(1,28)_ = 9.49, *p* = 0.005, such that the control group demonstrated a trending positive association between age and change in PSOC (*t* = 1.87, *p* = 0.084) and the FIND group showed a trending negative association (*t* = −1.856, *p* = 0.086). The group effect on other self-report measures was not significantly moderated by family variables (*p*s < 0.3).

Regarding the SST, group effects on change in SSRT showed a significant moderation by family income (square root transformed), *F*_(1,28)_ = 4.32, *p* = 0.047. Follow-up regression models by group indicated that mothers in the control condition had a non-significant positive association between income and change in SSRT (*t* = 1.731, *p* = 0.11), and FIND mothers did not (*t* = −0.489, *p* = 0.63). In the left IFG ROI, individual parameter estimates from change in the Correct Stop > Correct Go contrast over time were moderated by family income, *F*_(1,27)_ = 4.53, *p* = 0.043. The main effects of condition (*F* = 6.0, *p* = 0.021) and income (*F* = 4.95, *p* = 0.035) were also significant. Here, follow-up regression models by group revealed that the control group showed a trend-level positive effect of income on left IFG activity (*t* = 2.154, *p* = 0.052), but the FIND group did not (*t* = 0.613, *p* = 0.55). This effect was also seen with regard to maternal education, *F*_(1,27)_ = 8.44, *p* = 0.007, where the control group showed a trend-level positive effect of years of maternal education on left IFG activity (*t* = 2.17, *p* = 0.052), but the FIND group did not (*t* = −0.284, *p* = 0.781).

The significant group by time effect on percent DS traits endorsed was not significantly moderated by any of the family variables (*p*-values > 0.34). Collapsing across group, income (square root transformed) was significantly negatively correlated with parameter estimates of brain activity in the mPFC ROI from the Self > Change contrast, *r*_(32)_ = −0.396, *p* = 0.025. Visual inspection of the data indicated that mothers with a higher family income reported more of a drop in mPFC ROI activity from T1 to T2 compared to lower-income mothers. Lastly, there was a significant three-way interaction between maternal ACES, group assignment, and baseline mPFC activity from the Self > Change contrast for DS traits on change in PCDI, *F*_(2,25)_ = 4.262, *p* = 0.026. When this effect was decomposed by group, ACES had a significant negative influence on the association between mPFC activity and change in the PCDI in the FIND group (*t* = −3.963, *p* = 0.002), but not in the control group (*t* = 1.835, *p* = 0.091). In other words, mothers who reported experiencing high levels of childhood adversity demonstrated a stronger negative association between baseline mPFC activity and change in PCDI than lower-ACE mothers when participating in FIND, an association that did not exist in the control group. This may indicate that, for mothers who experienced adversity in their own childhoods, lower levels of baseline mPFC activity associated with thinking about one’s own DS parenting traits predicted greater improvements in self-reported difficult parent-child interactions over the course of FIND.

## Discussion

The purpose of the current study was to investigate the impact of the FIND video coaching program on two neurocognitive mechanisms hypothesized to underlie the program’s efficacy: inhibitory control and parenting self-evaluation. Utilizing a small sample of mothers with young children (ages four and under), we collected behavioral and brain data from functional neuroimaging tasks tapping each of these domains before and after the FIND program (or after waiting an equivalent amount of time in the control condition). At each visit, participating mothers performed experimental tasks while undergoing functional neuroimaging, then completed a set of surveys assessing positive and negative affect, parenting stress, and parenting sense of competency.

The data revealed that both hypothesized neurocognitive mechanisms were impacted by the FIND intervention, but in different domains. For inhibitory control, we found a significant group by time interaction in IC-related clusters in the left IFG and insula for the Correct Stop > Correct Go contrast. In both of these regions, this interaction was driven by an increase in activity in the FIND group but a decrease in the control group. This effect was moderated by measures of socioeconomic status (family income and maternal education, which were significantly correlated in this sample, *r* = 0.39, *p* = 0.027), such that there was a positive association between SES and IC behavior and left IFG activity for the control mothers but not those who participated in FIND. While the effect of FIND on IC behavior did not reach significance in this sample, it showed a strong trend in the hypothesized direction. Together, this suggests that the FIND program may buffer lower-income mothers from the SES-related decrease in IC seen in the controls.

The finding of improved inhibitory control in the FIND group parallels effects seen in prior studies that employ computer-based targeted inhibitory control training (e.g., Berkman et al., 2014). This suggests that FIND may be an ecologically valid way to train inhibitory control for parenting in this population, a claim which needs to be tested in a larger, higher-risk group of families. The achievement of this group level effect on brain activity across time is noteworthy given the once a week dose of FIND and aligns with theorizing in the cognitive training literature around the power of embedding the training of cognitive skills such as inhibitory control into salient real-world contexts (e.g., parent-child interactions).

Unlike IC, the effect of FIND parenting self-evaluation was only observed in the behavioral domain. There was a strong group by time interaction on percentage of child-supportive traits endorsed by mothers, where FIND mothers showed a marked increase in their endorsement of these terms while control mothers did not. This was not reflected in brain activity associated with the Self > Change contrast. We were surprised to find null effects of FIND on brain activity associated with the PSET, given the strong group effects on the endorsement of DS traits. One explanation for this result may be that, while FIND increases the number of DS traits caregivers identify with, this does not translate to a significant change in mPFC recruitment when these caregivers think about themselves as parents. The association between lower baseline levels of mPFC activity associated with DS trait endorsement and greater improvement in self-reported parenting stress, which exploratory analyses revealed may have been driven by mothers who experienced high levels of childhood adversity and who participated in FIND, suggests that this task may be a useful way of predicting who may report the greatest benefit of FIND on stress associated with difficult parent-child interactions.

Across both groups, change in mPFC activity was negatively correlated with family income, suggesting that higher-income mothers had more of a drop in mPFC activity associated with thinking about themselves as parents compared to lower-income mothers. This was not affected by the FIND intervention, which may indicate that, although FIND did shift parents’ endorsement of DS traits, perhaps it was not a strong enough identity-focused intervention to change the robust self-perceptions around parenting putatively indexed by mPFC activity.

On self-report measures of parenting stress and PSOC, there were null effects for FIND over time after controlling for state affect. These effects remained null when we did not control for state affect. One clear reason why we may not have found these effects in the present sample concerns the fact that, while many of these mothers were lower-income and had experienced early childhood adversity, this was a high-functioning sample. Many of these mothers reported participating because they were already deeply engaged in parenting support communities, interested in learning more about our research, and wanted to learn even more about parenting. In contrast, FIND was primarily designed for high-risk caregivers. As such, while the patterns were in the anticipated directions, the overall high level of parental investment reported by the present sample may have washed out the effects.

These results have a number of implications for interventions designed to enhance child-supportive parenting. First, as noted in the introduction, although prior studies have clearly demonstrated the ability to intervene on caregiver behavior, much less is known about how such interventions impact underlying brain functioning in areas relevant to parenting. Given the relatively brief nature of the FIND intervention, it was certainly possible that no effects from the neuroimaging data would have been detected. The fact that there were intervention effects, specifically on brain activity during the inhibitory control task, is therefore quite noteworthy. One of the most interesting aspects of this finding is that, unlike prior cognitive training studies that have used the same measure, FIND does not specifically target IC. The detection of intervention effects in the neuroimaging data provide justification for larger scale evaluation of FIND (and other interventions to enhance child-supportive parenting), with sufficient sample sizes to be able to conduct adequately powered mediation analyses.

The lack of main intervention effects on the brain-based measure of parenting self-evaluation (in spite of the observed effects of FIND on the behavioral PSET data) is also noteworthy. It is certainly possible that this is a domain that is less amenable to change (at least at a brain level). However, as noted previously, it also may be the case that the relatively high level of parenting competence in this sample created issues with restriction of range that would make detection of main effects less likely. To some extent, the presence of intervention effects on the PSET behavioral measure may justify ongoing investigation of this domain in future FIND evaluation work.

The exploratory moderation analyses yielded a number of findings suggesting that higher-risk mothers (e.g., higher adversity in childhood) may show greater behavioral and brain changes as a result of FIND. This is an interesting result because higher-risk samples can be more difficult to engage in family-based interventions (Korfmacher et al., 1999). In contrast, the positive, strength-based nature of FIND coaching may be especially well suited to higher-risk mothers. An alternative explanation for the moderation analysis results is that higher-risk mothers had more to gain from FIND due to lower levels of responsiveness at the outset. Regardless, these results highlight the importance in intervention work of examining for whom specific strategies are most effective.

There were several limitations of the present study. First and foremost, this was a preliminary study, limited by a small sample size. We were unaware of any similar effects in the literature on which to base a power analysis, and thus set our sample size based on budgetary constraints. The present results provide preliminary estimates of effect size for the effect of FIND on inhibitory control and parent self-evaluation, which should be pursued in a larger sample. Second, again with regard to the sample, this was a racially homogenous (90% Caucasian), English-speaking sample of biological mothers of children across a wide age range. While this impacts the generalizability of these findings, it may have also limited our ability to replicate the results of previous investigations of FIND on some of the variables chosen for this study. Third, it may be argued that there is a large gap between the forms of inhibitory control and parenting self-evaluation engaged in the MRI tasks and the way those phenomena are deployed during everyday parenting. As such, the effects documented here are likely weaker than if our tasks more closely simulated real-world parenting. However, this may make the fact that we found an effect of FIND on some of our hypothesized neurocognitive mechanisms all the more noteworthy. Finally, because we did not replicate the previous findings demonstrating a beneficial effect of FIND on parenting stress and sense of competency, we cannot definitively state that the changes in inhibitory control behavior and brain activity and parent self-evaluation behavior underlie the therapeutic effects of FIND. More work in a larger sample is needed to investigate these hypothesized associations. This work should also extend the present dependent variables to observed child-supportive parenting and/or serve and return behavior.

Despite these limitations, the present research provides important preliminary evidence about the neurocognitive mechanisms underlying the FIND video coaching program. Mothers of young children who participated in FIND demonstrated changes in brain activity associated with inhibitory control, as well as increased endorsement of child-supportive parenting traits. These effects were moderated by SES and maternal history of childhood adversity, suggesting that FIND may offer greater benefit for low-SES mothers and those who experienced early adversity. While more work is needed to investigate these findings in larger, and perhaps higher-risk samples, this study offers preliminary evidence of the behavioral and neural mechanisms of a targeted supportive parenting intervention.

## Data Availability

The datasets generated for this study are available on request to the corresponding author.

## Ethics Statement

This study was carried out in accordance with the recommendations of the University of Oregon Research Compliance Services Office with written informed consent from all subjects. All subjects gave written informed consent in accordance with the Declaration of Helsinki. The protocol was approved by the University of Oregon Institutional Review Board.

## Author Contributions

NG and PF conceived of and designed the study. NG collected the data. NG and KB analyzed the data. NG wrote the first draft of the manuscript. NG, KB, and LN wrote sections of the manuscript. All authors contributed to manuscript revision, read, and approved the submitted version.

## Conflict of Interest Statement

The authors declare that the research was conducted in the absence of any commercial or financial relationships that could be construed as a potential conflict of interest.

## Footnotes

^1^http://lcni.uoregon.edu/~jolinda/MRIConvert/

^2^http://www.fmrib.ox.ac.uk/fsl/

^3^http://www.fil.ion.ucl.ac.uk/spm/

^4^marsbar.sourceforge.net/
